# Ferritinophagy-Mediated Ferroptosis Involved in Paraquat-Induced Neurotoxicity of Dopaminergic Neurons: Implication for Neurotoxicity in PD

**DOI:** 10.1155/2021/9961628

**Published:** 2021-07-26

**Authors:** Yong Zuo, Jinhong Xie, Xincheng Li, Yan Li, Anand Thirupathi, Jianhua Zhang, Peng Yu, Guofen Gao, Yanzhong Chang, Zhenhua Shi

**Affiliations:** ^1^Laboratory of Molecular Iron Metabolism, College of Life Science, Hebei Normal University, Shijiazhuang, 050024 Hebei Province, China; ^2^Faculty of Sports Science, Ningbo University, Ningbo 315211, China

## Abstract

Parkinson's disease (PD) is a progressive nervous system disorder. Until now, the molecular mechanism of its occurrence is not fully understood. Paraquat (PQ) was identified as a neurotoxicant and is linked to increased PD risk and PD-like neuropathology. Ferroptosis is recognized as a new form of regulated cell death. Here, we revealed a new underlying mechanism by which ferritinophagy-mediated ferroptosis is involved in PD induced by PQ. The effect of PQ on movement injury in mice was investigated by the bar fatigue and pole-climbing test. SH-SY5Y human neuroblastoma cells were used to evaluate the mechanism of ferroptosis. Our results showed that PQ induced movement injury by causing the decrease in tyrosine hydroxylase in mice. In vitro, PQ significantly caused the iron accumulation in cytoplasm and mitochondria through ferritinophagy pathway induced by NCOA4. Iron overload initiated lipid peroxidation through 12Lox, further inducing ferroptosis by producing lipid ROS. PQ downregulated SLC7A11 and GPX4 expression and upregulated Cox2 expression significantly, which were important markers in ferroptosis. Fer-1, an inhibitor of ferroptosis, could significantly ameliorate the ferroptosis induced by PQ. Meanwhile, Bcl2, Bax, and p-38 were involved in apoptosis induced by PQ. In conclusion, ferritinophagy-mediated ferroptosis pathway played an important role in PD occurrence. Bcl2/Bax and P-p38/p38 pathways mediated the cross-talk between ferroptosis and apoptosis induced by PQ. These data further demonstrated the complexity of PD occurrence. The inhibition of the ferroptosis and apoptosis together may be a new strategy for the prevention of neurotoxicity or PD in the future.

## 1. Introduction

Parkinson's disease (PD) is the most frequent and debilitating group of motor disorders in the elderly population [1, 2] and has affected millions around the world from 1990 to 2015 [3]. The neuropathological features of PD are the loss of dopaminergic neurons in vulnerable brain regions, especially in the substantia nigra (SN) [4, 5]. The exact causes of PD are not well established. However, metabolic disorders of metal ions, oxidative stress, mitochondrial dysfunction, inflammation, and apoptotic processes have been implicated in PD [6–10]. Until now, there is no cure for PD because the molecular mechanism of PD has not yet been fully understood. An understanding of the mechanisms of PD may play an important role in the prevention and treatment of PD occurrence.

The disorder of metal ion metabolism, especially iron overload, is one of the important causes of PD. Iron deposition can induce toxicity of dopaminergic neurons and further promote the *α*-synuclein aggregation, which is the main neuropathological hallmark of PD [11–13].

Ferroptosis is recognized as a new form of regulated cell death which is initiated by severe lipid peroxidation relying on reactive oxygen species (ROS) generation and iron overload [14]. The main characteristics of ferroptosis include glutathione (GSH) peroxidase 4 (GPX4) inactivation or downregulation, inhibition of a specific light-chain subunit of the cystine/glutamate antiporter (SLC7A11 or system Xc-), and increased levels of free iron and lipid peroxidation [15, 16]. GPX4, as a key regulatory factor in ferroptosis, plays the main role in the repair of lipids injury and protecting membrane liquid using glutathione (GSH) as a cofactor [14, 16]. Nevertheless, the levels of GSH are mainly influenced by the function of SLC7A11. The inhibition of GPX4 and/or SLC7A11 can cause ferroptosis [16]. Since ferroptosis is closely associated with the incidence of many diseases (including neurodegenerative diseases, cancer, and diabetes), it has attracted an extensive attention as a target for the treatment of these diseases [15, 17, 18].

Paraquat (PQ), as a kind of pesticide in agriculture, is a widely used herbicide that was identified as a neurotoxicant and is linked to increased PD risk and PD-like neuropathology [19, 20]. PQ impairs the redox cycling of glutathione and thioredoxin which further affects the function to protect against oxidative stress in cells [21]. It was reported that iron enhanced PQ-mediated dopaminergic cell death [22] and PQ and iron exposure as possible synergistic environmental risk factors in PD [23], indicating that ferroptosis played an important role in PD induced by PQ. Although some mechanisms, including necrosis, apoptosis, and neuroinflammation, are involved in PQ-induced neurotoxicity [24–26], the exact mechanism of PQ-induced PD has not been fully understood. There are possible mechanisms that depict how ferroptosis inhibitors could be used for the treatment of PQ toxicity, but the direct experimental evidence is lacking. Here, we showed that PQ can significantly induce the neurotoxicity of dopaminergic neurons through the ferroptosis and ferritinophagy pathway, indicating that inhibition of the ferroptosis is a new strategy for the prevention of neurotoxicity or PD in the future.

## 2. Materials and Methods

### 2.1. Reagents

Paraquat dichloride (M813276) was purchased from Macklin. Ferrostatin-1 (Fer-1) (S7243) was purchased from Selleck Chemicals (Houston, TX, USA). Hoechest (H342), Mito-FerroGreen (M489), and FerroOrange (F374) were purchased from DOJINDO (Japan); antibodies of TH (ab112, 1 : 5000), FtL (ab109373, 1 : 10000), FtH (ab183781, 1 : 10000), and GPX4 (ab125066, 1 : 5000) were purchased from Abcam (USA); antibodies of FpN1 (MTPP11-S, 1 : 5000), DMT1+IRE (NRAMP21-S, 1 : 5000), and DMT1-IRE (NRAMP23-S, 1 : 5000) were purchased from ADI (USA); antibody of TfR1 (13-6800, 1 : 3000) was purchased from Invitrogen (USA); antibody of P-p38/p38 (#4511/#8690S, 1 : 2000) and Bax (#2772, 1 : 5000) was purchased from Cell Signaling Technology (USA). Antibody of Bcl-2 (GTX10064, 1 : 5000) was purchased from GeneTex (USA), and antibody of SLC7A11 (26864-1-AP, 1 : 5000) and COX2 (12375-1-AP, 1 : 2000) was purchased from Proteintech (Wuhan, China); antibody of 12Lox (sc-365194, 1 : 1000) was purchased from Santa Cruz Biotechnology; antibody of NCOA4 (ABP50693, 1 : 2000) was purchased from Abbkine (Wuhan, China).

### 2.2. Animal Experiments

Twenty male C57BL/6 mice at 12 weeks old were purchased from Hebei Medical University Experimental Animal Center. The animals were housed at room temperature with alternating 12 h light/dark cycles. Food and water were provided ad libitum. All experimental procedures involving animals and their care conformed to the guide for the care and use of laboratory animals of the National Veterinary Research and Quarantine Service of China. The administration of PQ in mice was according to the previously described methods [27]. Briefly, 10 mice of the experimental group were intraperitoneally injected with PQ (10 mg PQ (salt)/kg/dose) three times a week for 3 weeks according to the previous report [28]. Ten mice of the control group were intraperitoneally injected with the same dose of normal saline. Once the experimental schedule was completed, firstly, the animals were used for behavioral tests. Then, the mice were anesthetized with 0.4% pentobarbital sodium (1 mL/100 g) solution and perfused. The substantia nigra tissue was exfoliated for subsequent experiments.

### 2.3. Behavioral Test

For the bar fatigue test (rotating bar) as previously described [29], briefly, the mice were rotated at a constant 40 r/min speed. Each mouse was given three opportunities. The time that the mice fell off the bar was recorded as latency to fall. If the mice can hold for 5 min at any one of the three opportunities, the mice will be taken down from the rotating bar, and the recorded time was 300 s. If the mice could not hold for 5 min in three opportunities, data were recorded as the mean time on the rotating bar over the three test trials. Motor deficiency was evaluated according to the ability of the mouse to hold the rotating rod. The experiment of behavioral test was conducted for five consecutive days.

For the pole-climbing test as previously described [30], briefly, a rubber ball with a diameter of 3 cm is fixed on the top of a cylindrical wooden rod with a length of 60 cm and an inner diameter of 1 cm. The wooden rod is wrapped with gauze to prevent slipping. The mice were put on the rubber ball with head up, and the time was recorded till the mouse turns its head down and smoothly climbs down the wooden pole to the ground. Each mouse was tested three times. If the mouse stayed on the wooden pole for more than 120 s, it was recorded as the maximum value of 120 s. If the mouse falls off the pole, this score is not included.

### 2.4. Cell Culture and Treatments

Human neuroblastoma SH-SY5Y cells were grown in DMEM supplemented with 10% fetal bovine serum, 100 U/mL of penicillin, and 100 mg/mL of streptomycin. Cells were maintained at 37°C in a humidified 5% CO_2_ /95% air incubator. To investigate the neurotoxicity of PQ and the pathway of ferroptosis, we treated the SH-SY5Y cells with different concentrations of PQ for 24 h or treated the cells with Fer-1 (a type of inhibitor of ferroptosis) for 4 h and then exposed to PQ for 24 h to test the viability.

### 2.5. Assay of Cell Viability and Cell Morphology

Cell viability was investigated with a Cell Counting Kit-8 (CCK-8, MCE, China) according to the manufacturer's instructions. SH-SY5Y cells were seeded in 96-well plates. After treatment with or without PQ, 10 *μ*L of the CCK-8 solution was added to each well of the 96-well plates, and the cells were incubated at 37°C for 2–3 h. The absorbance was measured at 450 nm with a microplate reader (BioTek, USA). For the assessment of cell morphology, SH-SY5Y cells were examined using a phase-contrast light microscope (Olympus, Japan).

### 2.6. Glutathione (GSH), Glutathione Disulfide (GSSG), and NADP^+^/NADPH Assay

GSH and GSSG concentrations in SH-SY5Y cells were assessed by using the GSH assay kit and GSSG assay kit (purchased from Solabio, China). NADP+ and NADPH concentrations were tested by using assay kit (purchased from Beyotime, Shanghai, China) according to the manufacturer's instructions, respectively.

### 2.7. Immunohistochemistry Assay

For immunohistochemistry studies, after intraperitoneal injection of a 0.4% pentobarbital sodium (1 mL/100 g) solution, animals from each group were perfused with 0.9% saline, followed by perfusion with 4% paraformaldehyde in phosphate buffer (pH 7.4). The brains were carefully dissected, removed, postfixed, and then transferred to 30% sucrose for 2 days. The coronal brain sections were cut frozen with a thickness of 15 *μ*m, and then, the sections were washed with 0.01 M PBS three times for 5 min each time. The sections were incubated with 3% H_2_O_2_ for 20 min and then were washed with 0.01 M PBS three times for 5 min each time. The sections were then incubated with goat serum at 37°C for 60 min. The samples were incubated overnight at 4°C with the rabbit polyclonal antityrosine hydroxylase (TH) antibody. Next, they were incubated with biotinylated secondary goat anti-rabbit IgG at 37°C for 1 h. After being washed with PBS three times for 5 min each time, the sections were incubated in horseradish peroxidase- (HRP-) labeled streptavidin reagent for 1 h at room temperature and then were stained using a DAB kit (SK-4100; VECTOR, Kronshagen, Germany) for 40 s. Finally, the sections were dehydrated and mounted. Images were captured using a ZEISS LSM510 (LSM 510; Carl Zeiss, Oberkochen, Germany).

### 2.8. Transmission Electron Microscopy (TEM)

TEM assay was as previously our report [31]. Briefly, SH-SY5Y cells were fixed in 2.5% glutaraldehyde/2% paraformaldehyde (pH 7.2) overnight at 4°C. Samples were subsequently immersed in secondary fixative solution containing 1% osmium tetroxide in 0.1 M sodium cacodylate/HCl buffer (pH 7.2) for 4 h. Then, samples were dehydrated through 50%, 70%, 90%, 95%, and 100% alcohol, embedded with a mixture of resin and propylene oxide. Finally, the cells were in a 65°C oven for 48 h to polymerize. After polymerization, samples were sectioned at 100 nm, lifted onto 3 mm copper grids, and stained for 30 min in 1.5% aqueous uranyl acetate for 10 min in lead citrate. The samples were dried and viewed on the Hitachi H7000 TEM.

### 2.9. Terminal Deoxynucleotidyl Transferase-Mediated FITC-dUDP Nick-End Labeling (TUNEL) Assay

TUNEL detection was performed according to the manufacturer's instruction of TUNEL FITC Apoptosis Detection Kit (Vazyme Biotech CO., Nanjing China). Briefly, SH-SY5Y cells were seeded in a six-well plate at a density of 2.5 × 10^5^ cells per well and allowed to adhere in DMEM medium overnight. After washing with PBS, complete medium containing 0.8 *μ*M PQ was added into wells for 24 h. The nuclei were counterstained with DAPI, and the image was captured using a laser scanning confocal microscope (LSM 510; Carl Zeiss, Oberkochen, Germany).

### 2.10. Western Blot Analysis

The SN tissues of mice or SH-SY5Y cells were homogenized in RIPA buffer followed by centrifugation at 12,000 g for 20 min at 4°C. The supernatant containing proteins were collected and measured its content using protein quantification kit (Kang Wei, Beijing, China). The samples were resolved by 8–12% of SDS-PAGE, respectively, and then transferred to nitrocellulose membranes (Millipore, Bedford, MA, USA). The target proteins TH, FtH, FtL, FpN1, TfR1, DMT1 (+IRE), DMT1 (-IRE), NCOA4, SLC7A11, Cox2, GPX4, 12-Lox, Bcl2, Bax, P38, and P-p38 were detected by their primary antibodies. The relative expression quantity of proteins was normalized to that of *β*-actin (mouse monoclonal; Kang Wei, Beijing, China).

### 2.11. Detection of Intracellular ROS and Lipid ROS Levels

Intracellular ROS and lipid ROS levels were measured using previously described methods (Dixon et al., 2012). Briefly, after the treatment with or without PQ, intracellular levels of ROS and lipid ROS were examined using 2′,7′-dichlorofluorescein diacetate (H2DCF-DA, Thermo Fisher Scientific) and the BODIPY 581/591 C11 probe (Thermo Fisher Scientific), respectively. Cells were incubated with DCFH-DA (20 *μ*M) or BODIPY 581/591 C11 (10 *μ*M) for 30 min at room temperature in the dark, washed three times with PBS, and evaluated using an inverted microscope (IX83, Olympus, Japan) or a FACS Calibur flow cytometer (BD Biosciences, CA, USA) to measure ROS production.

### 2.12. Mitochondrial Membrane Potential (MMP) Assay

The MMP was measured by the MMP assay kit using JC-1 (Beyotime, Shanghai, China) as described previously. Briefly, the cells cultured with or without PQ on coverslips were incubated in 1 × JC − 1 in the medium for 20 min at 37°C. Cells are then precipitated by centrifugation (600 g) at 4°C. The fluorescence intensities were measured a FACS Calibur flow cytometer (BD Biosciences, CA, USA).

### 2.13. Assay of Iron Content

The iron content was assessed using FerroOrange and Mito-FerroGreen probes (DojinDo, Japan) for the measurement of cytoplasm iron and mitochondrial iron level, respectively. In the assay, ferric carrier proteins will dissociate ferric iron in the reductive environment. After reduction to the ferrous form (Fe2+), cytoplasm Fe2+ reacts with probes to produce a stable colored complex. The fluorescence intensity was observed using ZEISS LSM510 (LSM510; ZEISS, Germany).

### 2.14. Statistical Analysis

The quantitative experimental data were calculated as means ± standard deviation (SEM). Statistical analyses were performed using the GraphPad Prism 6 software (RRID: SCR_002798, San Diego, CA, USA). Statistical significance was determined using one-way or two-way ANOVA. *p* values < 0.05 were considered statistically significant.

## 3. Results

### 3.1. Effect of PQ on Nerve Tissue Injury in the Substantia Nigra (SN) and Striatum of Mice

**Figure 1:**
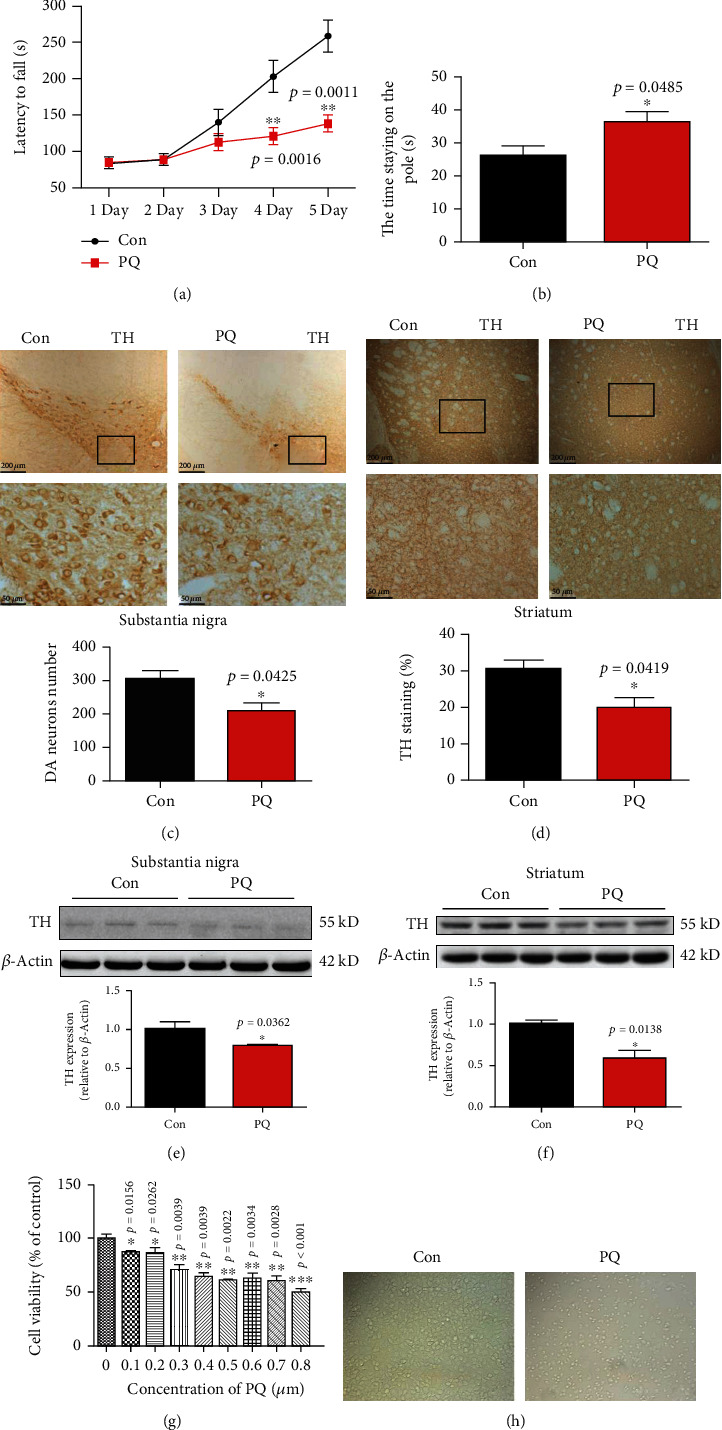
The effect of PQ on the neurotoxicity in substantia nigra and striatum in vivo and on SH-SY5Y cell viability in vitro. The mice were treated with PQ as described in Materials and Methods. The behavioral experiment was detected by the rotating bar and pole climbing. (a, b) PQ caused the dyskinesia in mice, and the data were calculated as the means ± SD (*n* = 10). (c–f) The effect of PQ on the expression of TH or number of dopaminergic (DA) neurons in substantia nigra and striatum. The data were calculated as the means ± SEM (*n* = 5). The statistical analysis was performed with one-way ANOVA. ^∗^*p* < 0.05 and ^∗∗^*p* < 0.01 compared with the control group. (g) Cell viability was measured by Cell Counting Kit-8 (CCK-8) assay. SH-SY5Y cells were incubated with or without different concentrations of PQ for 24 h. (h) The damage of 0.8 *μ*M PQ to cells was observed by morphology. Data were expressed as a percentage of the untreated control ± SEM (*n* = 8). ^∗^*p* < 0.05, ^∗∗^*p* < 0.01, and ^∗∗∗^*p* < 0.001 compared with the control group by ANOVA.

PQ is one of the common reagents used to make PD models. To confirm the success of PD model in mice, we did a behavioral test firstly. The results showed that the latency to fall and the time staying on the pole of PQ group were significantly different from that of the control group from the fourth day of testing as shown in Figures 1(a) and 1(b). Secondly, in order to verify the damage mechanism of PQ to nerve tissue in SN and striatum, we evaluated the effect of PQ on tyrosine hydroxylase expression and the number of dopaminergic neurons. The results showed that PQ significantly decreased the expression of TH and the number of dopaminergic (DA) neurons as shown in Figures 1(c)–1(f), indicating that PQ damaged the SN and striatum and caused the dopaminergic neurons death in vivo.

### 3.2. PQ Caused Dopaminergic Neurons Damage in a Dose-Dependent Manner

To further study the damage mechanism of paraquat on dopaminergic neurons in vitro, we used different concentrations of PQ to incubate the dopaminergic neurons SH-SY5Y for 24 h, then testing the cell viability. The results showed that PQ caused SH-SY5Y cells damage in a dose-dependent manner as shown in Figures 1(g) and 1(h). 0.8 *μ*M PQ reduced cell activity by about 50% compared with that of control group.

Therefore, 0.8 *μ*M was selected as the cell damage concentration for subsequent experiments.

### 3.3. PQ Stimulates Ferroptosis in SH-SY5Y Cells

**Figure 2:**
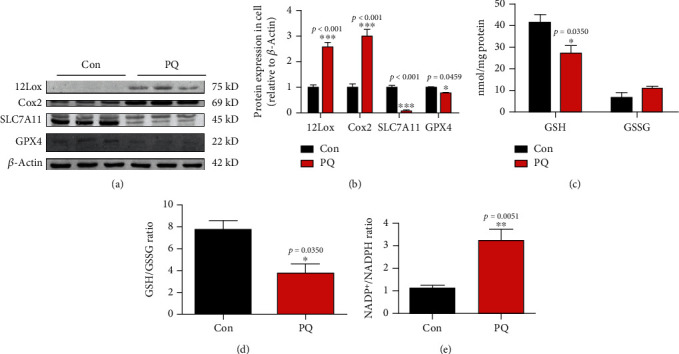
Effect of PQ on expression of the ferroptosis markers (12Lox, Cox2, SLC7A11, and GPX4), the contents or ratio of GSH and GSSG, and the ratio of NADP+/NADPH. (a, b) Cells were cultured with or without PQ for 24 h, and then, the proteins were extracted. 12Lox, Cox2, SLC7A11, and GPX4 in SH-SY5Y cell lysates were detected by western blot. Protein levels were also analyzed by calculating the ratio ± SEM (*n* = 5) of their band intensities over that of actin. ^∗^*p* < 0.05 and ^∗∗∗^*p* < 0.001 compared with control cells by ANOVA. (c–e) Cells were cultured with or without PQ for 24 h, and then, contents or ratio of GSH and GSSG and the ratio of NADP+/NADPH were detected by related kits as described in text. Data were expressed as a percentage of the untreated control ± SEM (*n* = 5). ^∗^*p* < 0.05, ^∗∗^*p* < 0.01, and ^∗∗∗^*p* < 0.001 compared with the control group by ANOVA.

Ferroptosis is recognized as a new form of regulated cell death. SLC7A11, GPX4, cyclooxygenase-2 (Cox2), and some lipoxygenase (Lox) are important markers during the process of ferroptosis [32]. To verify whether ferroptosis was involved in PQ-induced cell damage, we evaluated the expression of these markers. The results showed that PQ significantly downregulated the expression of GPX4 and SLC7A11 and upregulated the expression of 12Lox and Cox2 as shown in Figures 2(a) and 2(b). SLC7A11 is responsible for acquiring cystine from the extracellular environment and converting it to cysteine in the cytoplasm through a reduction reaction that consumes NADPH. Cysteine is then used to synthesize GSH. GPX4 depends on GSH for its enzyme activity. A growing number of studies have shown that SLC7A11-mediated cystine uptake plays a key role in inhibiting oxidative response and maintaining cell survival under oxidative stress conditions. Meanwhile, we found that PQ significantly decreased the levels of GSH and GSH/GSSG ratio and increased the ratio of NADP+/NADPH, as shown in Figures 2(c)–2(e). These findings revealed that PQ induced ferroptosis process in dopaminergic neurons.

### 3.4. PQ Caused the Cellular Iron Accumulation and Ferritinophagy in SH-SY5Y Cells

**Figure 3:**
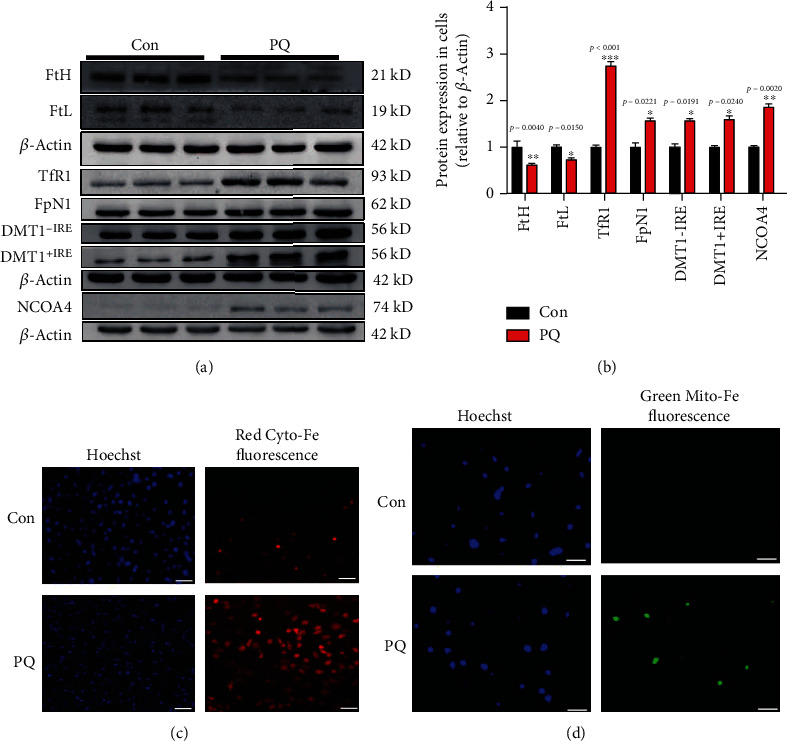
Effect of PQ on the expression of proteins related to iron metabolism, ferritinophagy-mediated marker NCOA4, and contents of iron in cytoplasm and mitochondrial. (a, b) Cells were cultured with or without PQ for 24 h, and then, the proteins related to iron metabolism (including FtH, FtL, TfR1, DMT1 (+IRE) and DMT1 (-IRE), and ferritinophagy-mediated marker NCOA4) were extracted and were detected by western blot. Protein levels were also analyzed by calculating the ratio ± SEM (*n* = 5) of their band intensities over that of actin. ^∗^*p* < 0.05, ^∗∗^*p* < 0.01, and ^∗∗∗^*p* < 0.001 compared with control cells by ANOVA. (c, d) Iron levels of cytoplasm and mitochondrial were detected by fluorescence probe as described in the text.

Intracellular iron homeostasis plays an important role in maintaining normal physiological function of the body. Intracellular iron homeostasis is tightly regulated by iron metabolism-related proteins including heavy ferritin (FtH), light ferritin (FtL), transferrin receptor1 (TfR1), divalent metal ion transporter1 with IRE (DMT1+IRE) or without IRE (DMT1-IRE), and FpN1. Therefore, we investigated the changes of these proteins and found that the expression levels of FtH and FtL, which were iron-storage proteins, were downregulated in PQ-treated SH-SY5Y cells. PQ also promoted the iron absorption by upregulated TfR1, DMT1 (+IRE), and DMT1 (-IRE) which were responsible for iron intake, meanwhile, promoting iron release by upregulating the expression of FpN1 which was the only characterized iron exporter in mammals as shown in Figures 3(a) and 3(b). Under normal physiological conditions, iron overload caused upregulation of ferritin expression and downregulation of TfR1 by the IRE/IRP pathway. Here, under administration of PQ, we wanted to know whether downregulation of ferritin expression meant lower intracellular iron levels. Therefore, we tested the iron contents in cytoplasm and mitochondria. The results showed that PQ significantly caused the iron accumulation in cytoplasm and in mitochondria as shown in Figures 3(c) and 3(d). Nuclear receptor coactivator 4 (NCOA4) mediated ferritinophagy is an autophagic phenomenon that specifically involves ferritin to release intracellular free iron and iron overload [33]. We found that PQ increased the iron levels through upregulating NCOA4 expression and initiating ferritinophagy pathway as shown in Figure 3(a).

### 3.5. PQ Promoted the Production of Intracellular ROS and Lipid ROS Production and Damage of Mitochondrial

**Figure 4:**
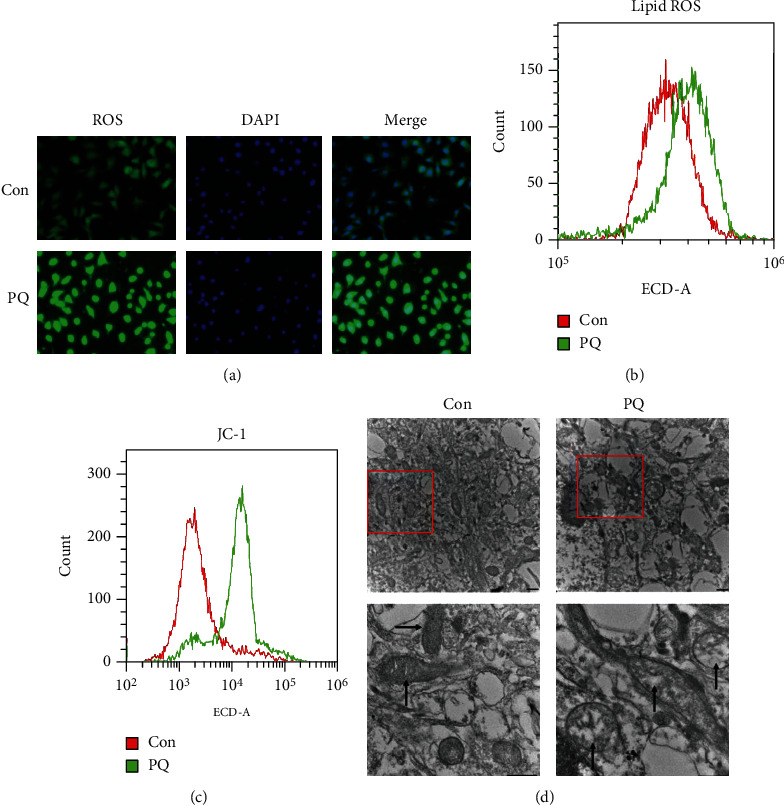
PQ increased the intracellular ROS and lipid ROS, decreased the MMP, and damaged the mitochondrial. Cells were cultured with or without PQ for 24 h, and then, the intracellular ROS (a), lipid ROS (b), and MMP (c) were tested by related kits as described in the text. (d) showed the effect of PQ on mitochondrial morphological damage under TEM.

Significant production of intracellular ROS and lipid ROS and impaired mitochondrial function are important features of ferroptosis. In order to further verify that ferroptosis involved in neurotoxicity induced by PQ, we investigated the intracellular ROS and lipid ROS production, MMP, and morphological changes in mitochondria in SH-SY5Y cells. Our results showed that cells exposed to PQ produced higher levels of intracellular ROS and lipid ROS compared with that of the control group as shown in Figures 4(a) and 4(b). Meanwhile, MMP of cells exposed to PQ also decreased significantly, and the integrity of mitochondrial morphology was destroyed as shown in Figures 4(c) and 4(d). These findings indeed reveal that PQ caused the ferroptosis in SH-SY5Y cells.

### 3.6. Inhibition of Ferroptosis Significantly Ameliorated Injury Induced by PQ

**Figure 5:**
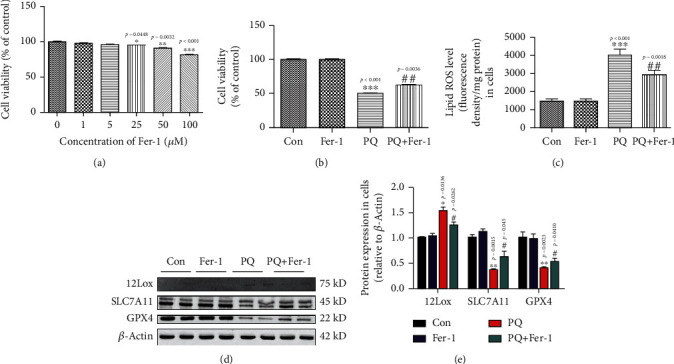
Inhibition of ferroptosis significantly ameliorated injury induced by PQ. Cells were cultured with different concentrations of Fer-1 for 24 h, and then, the cell viability was assayed by Cell Counting Kit-8 (CCK-8) assay (a). Cells were cultured with 5 *μ*M Fer-1 for 4 h, and then, the cells exposed to PQ together for 24 h, and then, cell viability was measured (b). Lipid ROS was tested by fluorescence probe as described in section of methods in the text (c). Data were expressed as a percentage of the untreated control ± SEM (*n* = 8). ^∗^*p* < 0.05, ^∗∗^*p* < 0.01, and ^∗∗∗^*p* < 0.01 compared with the control group by ANOVA. ^#^*p* < 0.05 and ^##^*p* < 0.01 compared with the PQ treatment group by ANOVA. (d, e) showed the effect of PQ on the expression of some ferroptosis markers including 12Lox, SLC7A11, and GPX4. Protein levels were also analyzed by calculating the ratio ± SEM (*n* = 5) of their band intensities over that of actin. ^∗^*p* < 0.05 and ^∗∗^*p* < 0.01 compared with control cells by ANOVA. ^#^*p* < 0.05 compared with the PQ treatment group by ANOVA.

Fer-1 is a specific inhibitor of ferroptosis. In order to test the effect of Fer-1 on cell viability of SH-SY5Y, we treated the cells with different concentrations of Fer-1 for 24 h. We found that higher concentration of Fer-1 had a damaging effect on cell viability, and no more than 5 *μ*M had no effect on cell viability as shown in Figure 5(a). We selected 5 *μ*M as inhibitor concentration of ferroptosis in the next experiment.

We treated the cells with Fer-1 (5 *μ*M) for 4 h in advance and then coincubated with PQ for 24 h. We also found that Fer-1 significantly ameliorated injury induced by PQ through inhibiting the production of lipid ROS as shown in Figures 5(b) and 5(c). Lox played an important role in lipid peroxidation and production of lipid ROS. We further verified that Fer-1 significantly inhibited the expression of 12Lox. In addition, more importantly, Fer-1 promoted the cystine input into cells through upregulation of SLC7A11 expression and suppressed the oxidative stress through upregulation of GPX4 under the administration of PQ as shown in Figures 5(d) and 5(e).

### 3.7. Inhibition of Ferroptosis Alleviated Mitochondrial Damage Induced by PQ

**Figure 6:**
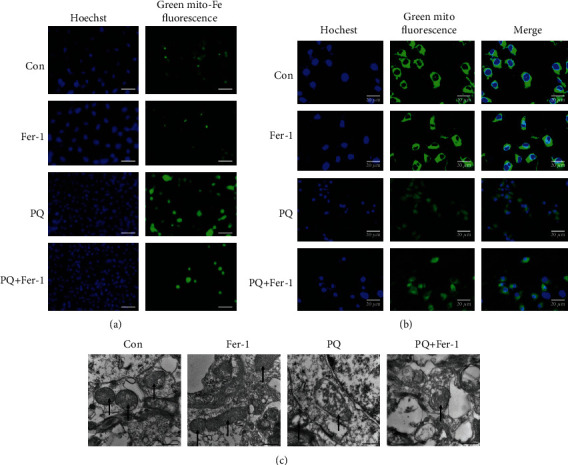
Inhibition of ferroptosis alleviated mitochondrial damage induced by PQ. Cells were cultured with 5 *μ*M Fer-1 for 4 h, and then, the cells exposed to PQ together for 24 h, and then, the iron contents of mitochondrial were measured (a). (b, c) showed the mitochondrial morphology changes after Fer-1 inhibiting ferroptosis induced by PQ.

Mitochondrial function is closely related to ferroptosis. We had confirmed that PQ caused the iron overload in mitochondrial which maybe further damaged the function of mitochondrial through Fenton reaction. To test whether inhibiting ferroptosis could prevent iron increase in mitochondria and thus alleviate PQ-induced mitochondrial functional impairment, we further assay the iron levels in mitochondrial. We found that the fluorescence intensity of cells treated with Fer-1 and PQ together significantly was lower than that of cells treated with PQ alone, indicating that inhibition of ferroptosis prevented the iron increase in mitochondrial as shown in Figure 6(a).

The more complete the mitochondrial morphology is, the closer the combination with mitochondrial tracker is. We used fluorescence probe and transmission electron microscopy to observe the morphological changes of mitochondria as shown in Figures 6(b) and 6(c). The results showed that inhibition of ferroptosis significantly alleviated mitochondrial damage induced by PQ.

### 3.8. Bcl2/Bax and Phospho-p38 Mitogen-Activated Protein Kinases (P-p38) Pathway Mediated the Cross-Talk between Ferroptosis and Apoptosis Induced by PQ

**Figure 7:**
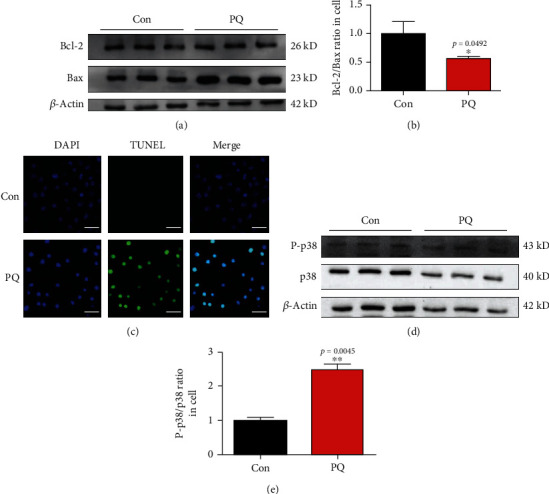
PQ induced apoptosis through P-p38 and Bcl2/Bax pathways. Cells were cultured with or without PQ for 24 h, and then, proteins related apoptosis (including Bcl2, Bax, P-p38, and P38) were extracted and then were detected by western blot (a, b, d, e). (c) showed the apoptosis induced by PQ detected by TUNEL methods as described in the text.

Although ferroptosis is considered a distinctive form of cell death compared to other types of death such as apoptosis, some reports showed that ferroptosis interplayed with apoptosis [34, 35]. In order to find which pathway mediated the cross-talk between ferroptosis and apoptosis induced by PQ, we evaluated the expressions Bcl2, Bax, P-p38, and p38. Our results showed that the ratio of Bcl2/Bax of the PQ treatment group significantly decreased compared with that of control group. On the contrary, the ratio of P-p38/p38 of the PQ treatment group significantly increased compared with that of control group as shown in Figures 7(a), 7(b), 7(d), and 7(e). TUNEL assay also proved that PQ caused the increase in apoptotic body significantly. These data indicated that Bcl2/Bax and phospho-p38 mitogen-activated protein kinases (P-p38) pathway mediated the cross-talk between ferroptosis and apoptosis induced by PQ.

## 4. Discussion

PD, as the second most common neurodegenerative disease after Alzheimer's disease (AD), is pathologically characterized by the loss of nigrostriatal dopaminergic innervation [36, 37]. In addition to genetic factors, environmental factors such as PQ, rotenone, and 1-methyl-4-phenyl-1,2,3,6-tetrahydropyridine (MPTP) also caused nigrostriatal dopaminergic death and play an important role in the development of PD [38–41]. However, the exact mechanism of the cell death is not clear fully understand.

In the present study, we found a new potential mechanism of PQ-induced neurotoxicity, that is ferritinophagy-mediated ferroptosis involved in damage of dopaminergic neurons. Firstly, we used PQ to make PD model in mice successfully. We found that PQ caused the bradykinesia which was neuropathological features of PD through decreasing TH content and number of dopaminergic neurons in SN and striatum as shown in Figure 1. Secondly, we investigated the hypothesis that ferroptosis was involved in neurotoxicity induced by PQ in PD cell model. We treated the SH-SY5Y cells with 0.8 *μ*M PQ for 24 h; we found that the cell viability decreased significantly, indicating that dopaminergic neurons SH-SY5Y were dramatically damaged as shown in Figure 1.

Ferroptosis was a form of regulated cell death characterized by the iron-dependent accumulation of lipid hydroperoxides to lethal levels and had been implicated in the pathological cell death associated with degenerative diseases [42] Lipoxygenase- (Lox-) mediated generation of lipid peroxides enhanced ferroptosis induced by erastin and RSL3 [43]. SLC7A11 functioned to import cystine for GSH biosynthesis [44]. GSH was GPX4 coenzyme. Cox2 is induced in cells undergoing ferroptosis [32]. Therefore, Lox, SLC7A11, GPX4, and Cox2 were all the important markers of ferroptosis. In order to further identify the effect of ferroptosis on neurotoxicity induced by PQ, we evaluated the expression of these proteins. The results showed that the expression of 12Lox and Cox2 upregulated and GPX4 downregulated in SH-SY5Y cells exposed to PQ (Figures 2(a) and 2(b)). Meanwhile, the contents of GSH and NADPH which was responsible for supplying H to GSSG to convert GSSG to GSH decreased significantly (Figures 2(c)–2(e)). These data indicate that PQ caused ferroptosis in SH-SY5Y cells. Iron homeostasis played an important role in maintaining cellular function. Systemic and cellular iron metabolism is mediated by iron intake, storage, utilization, and efflux and regulated by multiple proteins including ferritin, TfR1, DMT1, and FpN1 [45]. Excess iron usually was stored in ferritin to eliminate the toxicity of iron. TfR1 and DMT1 were responsible for transporting iron into cells. FpN1 is the only known iron efflux pump in vertebrates. Under normal physiological conditions, these protein expressions were regulated by IRE/IRP system. When iron levels increased, TfR1 expression would upregulate, and ferritin would downregulate. In recent years, more and more evidence shows that iron overload is an important risk factor for PD [11, 46, 47]. Excessive iron is a key factor in initiating ferroptosis [14, 48]. Here, we showed that PQ upregulated the expression of TfR1, DMT1, and FpN1, indicating that the turnover rate of iron into and out of cells is increased. Meanwhile, we found that FtH and FtL downregulated which meant that iron levels seemingly decreased as shown in Figures 3(a) and 3(b). In order to verify whether PQ induced the iron increased, we identified the ferrous ions in cytoplasm and mitochondrial by fluorescence staining. The results showed that PQ indeed caused a significant increase in cellular iron as shown in Figures 3(c) and 3(d), indicating that iron levels may not be regulated by the IRE/IRP system under abnormal physiological conditions. Li reported that LPS leads to ferritinophagy and ferroptosis of cardiomyocytes through increasing NCOA4 expression [49]. Here, we further showed that increased cellular iron contents were associated with ferritinophagy through upregulating NCOA4 as shown in Figure 3(a). Iron accumulation initiated the large lipid ROS production which is one of the major characteristics of ferroptosis and caused the mitochondrial function damage as shown in Figure 4.

Fer-1 was a specific inhibitor of ferroptosis. We showed that when SH-SY5Y cells were treated with PQ and Fer-1 together, Fer-1 could inhibit cell injury by suppressing lipid ROS production and ferroptosis through upregulating expression of GPX4 and SLC7A11 as shown in Figure 5. Meanwhile, Fer-1 also inhibited the ferrous iron accumulation in mitochondrial and protected against the PQ-induced mitochondrial damage, keeping the integrity of mitochondrial shown in Figure 6. The function of mitochondria is closely related to apoptosis and ferroptosis [50, 51]. Ferroptosis was primarily characterized by condensed mitochondrial membrane densities and smaller volume than normal mitochondria, as well as the diminished or vanished of mitochondria crista and outer membrane ruptured [14]. We observed that mitochondrial morphological characteristics induced by PQ did not quite fit that description. Previous studies had shown that PQ could induce cellular apoptosis [52, 53]. Our data suggested that there was some cross-talk between apoptosis and ferroptosis. Here, we found that Bcl2/Bax and phospho-p38 mitogen-activated protein kinases (P-p38) pathway mediated the cross-talk between ferroptosis and apoptosis induced by PQ, indicating that PQ-induced neurotoxicity was achieved through multiple pathways.

## 5. Conclusions

**Figure 8:**
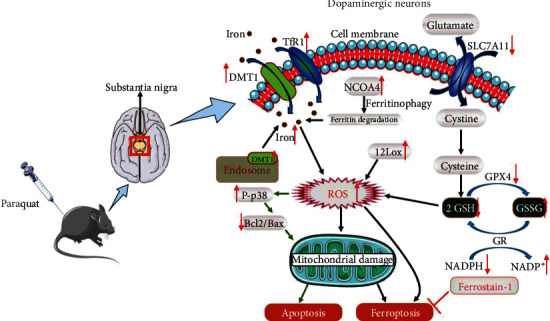
Schematic diagram of the neurotoxicity effect of PQ through cross-talk between ferritinophagy-mediated ferroptosis and apoptosis.

In conclusion, we found that ferritinophagy-mediated ferroptosis was involved in PQ-induced neurotoxicity of dopaminergic neurons. Bcl2/Bax and P-p38 pathways mediated the cross-talk between ferroptosis and apoptosis induced by PQ. The schematic diagram of the neurotoxic effect of PQ in mice is shown in Figure 8. Inhibition of the ferroptosis and apoptosis together may be a new strategy for the prevention of PD in the future.

## Data Availability

The datasets are available from the corresponding author on reasonable request.

## Abbreviations

PQ: Paraquat dichloride
PD: Parkinson's disease
GSH: Glutathione
GSSG: Glutathione disulfide
GPX4: Glutathione peroxidase 4
SLC7A11: Cystine/glutamate antiporter
TfR1: Transferrin receptor 1
FtH: Heavy ferritin
FtL: Light ferritin
ROS: Reactive oxygen species
MMP: Mitochondrial membrane potential
NADPH: Nicotinamide adenine dinucleotide phosphate
Fer-1: Ferrostatin-1
COX2: Cyclooxygenase-2
12-LOX: 12-Lipoxygenase
NCOA4: Nuclear receptor coactivator 4
DMT1: Divalent metal transporter 1
FpN1: Ferroportin 1
SN: Substantia nigra.
